# The Readability Study: A Randomised Trial of Health Information Written at Different Grade Reading Levels

**DOI:** 10.1007/s11606-024-09200-z

**Published:** 2024-12-20

**Authors:** Olivia Mac, Julie Ayre, Kirsten McCaffery, Farzaneh Boroumand, Katy Bell, Danielle M. Muscat

**Affiliations:** 1 Sydney School of Public Health, Faculty of Medicine and Health, Sydney Health Literacy Lab, The University of Sydney, Sydney, NSW 2006 Australia; 2 Sydney School of Public Health, Faculty of Medicine and Health, The University of Sydney, Sydney, Australia

**Keywords:** health literacy, readability, health communication

## Abstract

**Background:**

Despite increasing attention on health literacy and the inclusion of grade reading level recommendations in guidelines, it remains unclear if lowering the grade reading level of written health information to specific target grades improves patient-related outcomes.

**Objective:**

To assess whether grade reading level of written information affects knowledge, perceived reading ease, acceptability and trustworthiness of information and, to explore whether information written at a lower grade reading level reduces disparities in outcomes across health literacy levels.

**Design:**

We conducted a 4-arm online randomized trial with a community sample of adults living in Australia from 31 July to 20 September 2023.

**Experimental Arms:**

Participants were randomised to one of four arms: Information about sciatica and knee osteoarthritis written at a grade 8, 10, 12 or 14 reading level. Readability was assessed using the SMOG Index and iteratively revised to each lower grade.

**Measures:**

Primary outcome was knowledge of health conditions. Secondary outcomes were brief knowledge, perceived reading ease, acceptability (i.e., perceived usefulness and likelihood to recommend) and trustworthiness of information.

**Results:**

2235 participants were randomised and included in the analysis. Mean age was 41 years and 54.5% identified as female. Low health literacy was identified in 28.2% of participants. We found no evidence of a main effect of grade reading level on knowledge (grade 8: 9.0 (SD = 2.7), grade 10: 9.1 (SD = 2.6), grade 12: 8.9, grade 14: 9.1 (SD = 2.7). Participants with high health literacy had higher knowledge scores overall, however, there was no evidence that health literacy modified the effect of grade reading level. There were no significant differences in any of the secondary outcomes.

**Conclusions:**

Our study showed no difference in knowledge when grade reading level was manipulated alone. Our findings indicate there is limited value in reducing grade reading level without attention to other health literacy principles.

**ANZCTR trial registry number:**

ACTRN12623000224628p.

## INTRODUCTION

The importance of ensuring health information that can be understood and used by people from diverse backgrounds and varying levels of health literacy is widely recognised. Across the world, many health communication policy documents and guidelines recommend the use of health literacy tools and strategies to ensure the accessibility of written health information.^1–4^ There is convincing evidence that elements of health-literate document design collectively can improve health outcomes such as comprehension, behavioural intent and health-related skills.^5,6^ Health literacy describes the personal competencies and organisational structures, resources and commitment which enable people to access, understand, appraise and use information in ways that promote and maintain good health.^7^ Low health literacy is associated with a range of negative health outcomes including increased hospitalizations and poorer ability to interpret health messages.^8^ While health literacy is associated with educational attainment, it has been shown to have an independent impact on health outcomes.

Readability is an objective measure of text difficulty and is one of the most commonly approaches to develop, evaluate and revise written health information.^9^ It is operationalised by mathematical formulas which use features of text such as sentence and word length to calculate a readability score, usually in the form of a school grade reading level. One widely used formula in health applications is the Simple Measure of Gobbledygook (SMOG) Index^10^which uses the number of polysyllabic words to calculate a readability score. The SMOG index is considered more robust and less likely to underestimate the grade reading level compared to other formulas.^11^ Readability assessments are easy to administer, with many readily available software that can calculate replicable and objective estimates within seconds.^12^ Grade reading level recommendations may serve as a useful benchmark when revising written health information. Unlike other health literacy tools, readability scores can also readily be used for evaluation and monitoring at scale.

Guidelines often include recommendations for health information to be written at specific grade reading levels. These recommendations can vary across organisations and countries. In the United States, the Agency for Healthcare Research and Quality recommend grade 5–6 or below for adults with low health literacy.^1^ In the United Kingdom, the National Health Service recommend a grade reading level between 6 and 9^13^ and in Australia, two state health departments recommend a grade 8 reading level.^4,14^ Importantly, the rationale behind the designation of grade reading level thresholds is not transparent and the evidence supporting choice of threshold is unclear.

While there is some promising evidence that reducing grade reading level of health information can contribute to improved health outcomes,^15–18^ few studies have rigorously evaluated its unique contribution. Most previous studies have evaluated complex interventions; revising the text’s grade reading level was only one component of text simplification.^19–21^ Often, studies only compared two grade reading levels, with the gap between the standard and simplified texts ranging from 2–13 grade levels. Many studies used the Flesch Kincaid Grade Level^22^ to assess readability, however, research has demonstrated that this formula can underestimate the grade reading level by 2–3 grades.^11,12^ Similarly, there is a lack of reporting on the methods of calculating readability assessments. This has important implications as readability scores can vary substantially depending on the online calculator used.^12^ In addition, there were substantial differences in the comparisons made across studies. These factors make it difficult to draw accurate conclusions and determine the true effect of reducing the grade reading level of written health information.

In practice, there can be too much focus on revising health information to meet these potentially arbitrary grade reading level recommendations. Reducing the grade reading level of written health information can be time and resource intensive especially to achieve lower levels (e.g., grade 6–8), as it may impair cohesion and risk the removal of key messages. In order for consumers to effectively act on health information, it is important that it is considered both acceptable and trustworthy. Perceptions of trustworthiness and acceptability may vary depending on both the health literacy demands of information as well as the health literacy level of the reader.^23,24^

There is a need to develop health information that is understandable and accessible to people with varying levels of health literacy. However, issues may arise if this is at the expense of acceptability and trust. It is, therefore, important to ensure that reaching target grade reading levels is worthwhile and improves patient-related outcomes.

We conducted a randomised trial to evaluate the effect of health information written at different grade reading levels on knowledge, perceived reading ease, acceptability and trust. Our objective was to inform the development of written health information that optimises consumer understanding.

## MATERIALS AND METHODS

### Study Design

This was a four-arm randomised trial delivered online via Qualtrics. The trial was prospectively registered with the Australian New Zealand Clinical Trial Registry (ACTRN12623000224628p) and approved by the University of Sydney Human Research Ethics Committee (Protocol number 2023188).

### Participants and Recruitment

A community sample of adults aged 18 and over living in Australia were invited to participate in the study between July and September 2023. To be eligible, participants needed to be able to read, understand and give informed consent in English. Participants were recruited through Dynata, an online market research company experienced in panel survey sampling. Dynata have extensive online panels of participants who complete research in exchange for points that can be redeemed for rewards (such as gift vouchers, cash or donations to charity). We used quota sampling to recruit 50% of participants with less than university education and equal numbers of men and women.

### Randomization

Participants were randomised to one of the four experimental arms: health information written at a grade reading level of 8, 10, 12 or 14. The Qualtrics randomiser utilises the Mersenne Twister pseudorandom number generator to create allocation sequences. Participants and survey administers were blinded to the group allocation at the time of randomization.

### Procedure

**Figure 1:**
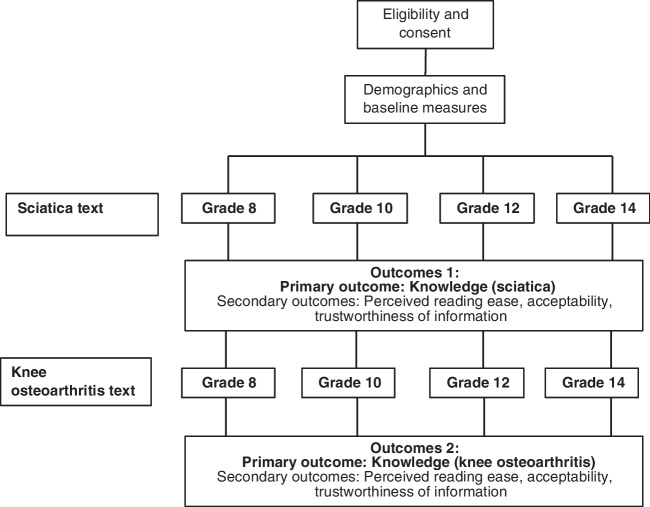
Study design.

After providing their informed consent, participants completed a commitment check, where participants were asked if they committed to providing thoughtful answers. Those responding ‘no’ or ‘I cannot commit either way’ were excluded. Participants were then directed to complete screening, demographic and baseline measures. Health literacy was measured using the widely used single item literacy screener^25^ as well as a performance-based measure based on comprehension of a fictitious medicine label^26^ Self-reported and performance-based knowledge of sciatica and knee osteoarthritis were also assessed at baseline. See Supplementary Table 1 for further detail. Participants then completed a distractor task to mitigate recall bias and an attention check to screen out careless respondents. Participants that failed the attention check were excluded from the analysis. Participants were randomised to one of the four experimental arms and presented with two pieces of health information written at the same grade reading level (within each randomised group). The order that participants saw the two topics was randomized. Outcomes (knowledge, perceived reading ease, acceptability and trust) were measured immediately after participants read each text. See Fig. 1.

### Experimental Arms

The experimental arms were health information written at one of four grade reading levels. Grade 8 was selected as the lowest level based on Australian and international readability recommendations.^4,13^ As public-facing health information is often written at a grade 12–14 reading level,^27,28^ we selected grade 14 as the upper limit. The trial texts were adapted from UpToDate: Patient Education: Low back pain in adults (beyond the basics)^29^ and UpToDate: Patient Education: Osteoarthritis Treatment (beyond the basics).^30^ UpToDate is a reputable source of written health information and patient education materials. We extracted sections of the material pertaining to overview of the condition, symptoms, management options and the effectiveness of different management options. Texts were revised slightly to meet a grade 14 reading level and to ensure they clearly included content covered in the validated knowledge items. The grade reading level of the material was assessed using the Sydney Health Literacy Lab (SHeLL) Health Literacy Editor^31^ which uses the SMOG Index,^10^ to calculate the readability score. The SMOG index uses the number of polysyllabic words (words with three or more syllables) and the number of sentences to calculate the approximate grade reading level of the text. The SMOG Index is a widely used and robust formula^11^ and can be reliably estimated using the Health Literacy Editor.^12^ The grade 14 text was iteratively revised down to each lower grade reading level by reducing the sentence length, replacing polysyllabic words with shorter words and converting text into bullet points where feasible. In order to isolate the effect of reducing the grade reading level, no diagrams or graphics were added, and no changes were made to the length of content, key messages, tone or font size. We made only minimal changes to the layout and organisation when necessary (e.g., addition of bulleted text). Texts were revised by OM with input from JA, DM, KB and KM.

### Measures

Baseline and outcome measures are described in Table 1. We used a combination of previously published, validated measures and purpose-built measures developed by the research team. Primary and secondary outcomes were measured immediately after reading each text. Knowledge items and health texts were piloted with consumers and academic researchers with expertise in the relevant conditions to ensure understandability, retention of key messages, clinical accuracy and to avoid ceiling or floor effects (i.e., to ensure appropriate task difficulty).

**Table 1:** Description of Measures

	Measure	Time point	
		Pre	Post
Health literacy: self-report	Validated single item literacy screener^25^ to identify people with low health literacy. Participants are asked ‘*How confident are you filling out medical forms by yourself.’* The item is rated on a 5-point response scale ranging from ‘not at all’ to ‘extremely.’ The threshold for inadequate health literacy is ‘somewhat’ or less	X	_
Health literacy: performance-based	Performance based measure of functional health literacy developed by Bostock and Steptoe ^26^. Participants are asked to read a fictitious medicine label and answer four comprehension questions such as ‘*what is the maximum number of days you can take this medicine’* and ‘*list one situation for which you should not take this medicine’.* This measure was developed according to a conceptual framework that defines health literacy as the ability to fulfil goal-directed tasks. Health literacy is categorised as high (all correct), medium (one incorrect response), and low (more than one incorrect response)	X	_
Primary outcome			
Extended knowledge	For each topic, knowledge was assessed using 4 items adapted from validated measures and 3 purpose-built items. Knowledge scores for each topic were summed to obtain a total knowledge score out of 14 Validated items: Four questions assessing knowledge of sciatica adapted from the validated Decision Quality Worksheet: Treatments for herniated disc. ^32^ Questions included yes/no response options e.g., *‘Can exercise make sciatic pain feel better’* and multiple-choice questions e.g., ‘W*hat treatment is most likely to provide faster relief from sciatic pain.’* Four true/false questions assessing knowledge of knee osteoarthritis adapted from the validated Osteoarthritis Knowledge Scale (OAKS).^33^ For example, *‘Osteoarthritis symptoms only get worse over time.’* Purpose-built items: Three purpose-built questions for each topic based on the key messages of the text e.g., *‘Imaging is recommended for most people with sciatica’* and ‘*If you have knee osteoarthritis, there are things you can do to…:’*	_	X
Secondary outcomes			
Brief knowledge	Four items for each topic adapted from validated scales ^32,33^ (see extended knowledge). Knowledge scores from both topics summed to obtain a total brief knowledge score out of 8	X	X
Perceived reading ease	Two items adapted from previous research using a 7-point Likert scale. ^41^ Participants were asked to indicate how easy or difficult the information was to read (1 = difficult to read, 7 = easy to read) and how straightforward or confusing they found the information (1 = confusing, 7 = straightforward)	_	X
Acceptability	Two purpose-built items assessed using a 5-point Likert scale. Participants were asked to indicate how strongly they agreed with the following statements: *If I was looking for information about [sciatica or knee osteoarthritis] I would have found this useful,* and ‘*I would give this information to a friend if they wanted to know more about [sciatica or knee osteoarthritis]* (1 = strongly disagree, 5 = strongly disagree)*.* Scores were averaged across the two items and topics	_	X
Trustworthiness of information	Single purpose-built item. Participants were asked to indicate how trustworthy they found the information on a scale of 1 *(not at all trustworthy*) to 10 (*very trustworthy*). Scores averaged across the two topics	_	X

### Primary Outcome

The primary outcome was knowledge, measured using a total of 14 items: Four validated and three purpose-built items for each topic. Validated knowledge (which included the four validated items for each topic only) was assessed as a secondary outcome (see below). For sciatica, the validated items were adapted from the Treatments for Herniated Disc – Decision Quality Instrument.^32^ For knee osteoarthritis, the validated items were adapted from the Osteoarthritis Knowledge Scale (OAKS).^33^ Purpose-built items were developed based on the key messages of each text and revised with input from experts and consumers. Scores were summed across the two topics to obtain a final extended knowledge score (number of correct responses out of 14).

#### Secondary Outcomes

Secondary outcomes included validated knowledge, perceived reading ease, acceptability and trustworthiness of information.

### Statistical Analysis

A sample size estimate of 2200 was based on 95% power at an alpha of 0.05% to detect a small effect size. Descriptive statistics (frequency for categorical variables and mean [standard deviation (SD)] for continuous variables) of sociodemographic and health characteristics, and outcome measures were calculated using R version 4.2.3.^34^ The primary and secondary outcomes from both conditions were pooled for analysis, as we expect the effects of readability to generalise across health conditions.

Multiple linear regression was conducted to explore the effect of grade reading level on the primary outcome, controlling for age, education, language spoken at home, baseline performance-based knowledge and self-reported knowledge. A multiple linear regression was also used to investigate the impact of health literacy and education level on knowledge scores across the four grade reading levels and to test for an interaction between health literacy and grade reading level (condition).

## RESULTS

**Figure 2:**
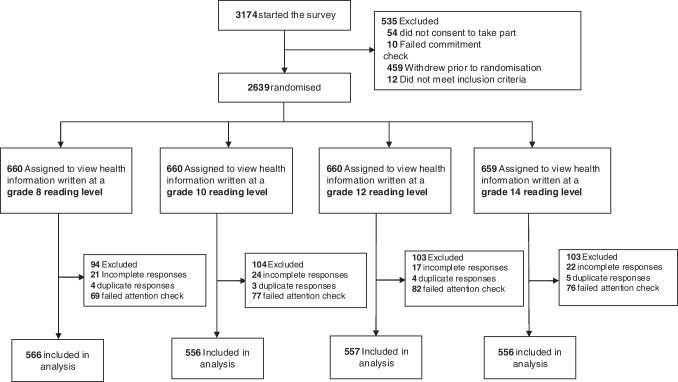
Participant flow diagram.

Of the 3174 people who opened the survey link, 2639 consented to take part in the study and were randomised to one of the four study arms (Fig. 2). 2235 (85%) provided complete responses that were deemed valid and were included in the final analysis.

Descriptive characteristics of the analysis sample are presented in Table 2. The mean age was 41 years and 54.5% identified as female. Most participants (93.1%) reported speaking English at home. Inadequate health literacy was observed in 9.4% of the sample using the Single Item Literacy Screener. Using the performance-based measure of health literacy, 28.2% of the sample were identified as having low health literacy.^26^

**Table 2:** Participant Characteristics of the Analysis Sample (*N* = 2235), by Randomised Intervention Group

	No. (%)				
	Grade 8 (*n* = 566)	Grade 10 (*n* = 556)	Grade 12 (*n* = 557)	Grade 14 (*n* = 556)	Total (*n* = 2235)
Age group					
18–29	141 (24.9)	129 (23.2)	131 (23.5)	141 (25.4)	542 (24.3)
30–39	205 (36.2)	198 (35.6)	207 (37.2)	182 (32.7)	791 (35.4)
40–49	44 (7.8)	56 (10.1)	55 (9.9)	58 (10.4)	213 (9.5)
50–59	44 (7.8)	35 (6.3)	38 (6.8)	35 (6.3)	152 (6.8)
60–69	58 (10.2)	72 (12.9)	59 (10.6)	61 (11.0)	250 (11.2)
70 and older	74 (13.1)	66 (11.9)	67 (12.0)	79 (13.2)	286 (12.8)
Gender					
Male	266 (47.0)	245 (44.1)	259 (46.5)	233 (41.9)	1003 (44.9)
Female	297 (52.5)	310 (55.8)	296 (53.1)	321 (57.7)	1224 (54.8)
Other	3 (0.5)	1 (0.2)	2 (0.4)	2 (0.4)	8 (0.4)
Educational level					
Bachelor’s degree or above	255 (45.1)	265 (47.7)	286 (51.3)	245 (44.1)	1051 (47.0)
Diploma or certificate	110 (19.4)	114 (20.5)	117 (21.0)	124 (22.3)	465 (20.8)
Trade apprenticeship	35 (6.2)	40 (7.2)	39 (7.0)	39 (7.0)	153 (6.8)
Higher school certificate	95 (16.8)	93 (16.7)	76 (13.6)	110 (19.8)	374 (16.7)
School certificate	58 (10.2)	37 (6.7)	30 (5.4)	34 (6.1)	159 (7.11)
No school or other qualification	13 (2.3)	7 (1.3)	9 (5.4)	4 (0.7)	33 (1.5)
Aboriginal and/or Torres Strait Islander origin					
Yes	33 (5.8)	14 (2.5)	30 (5.4)	30 (5.4)	107 (4.8)
No	530 (93.6)	523 (93.9)	533 (93.9)	534 (94.2)	2114 (94.6)
Not stated	3 (0.5)	5 (0.9)	4 (0.7)	2 (0.4)	14 (0.6)
Country of birth					
Australia	444 (78.4)	419 (75.4)	425 (76.3)	426 (76.6)	1714 (78.7)
Other	122 (21.6)	137 (24.6)	130 (23.4)	130 (23.4)	521 (23.3)
Main language spoken at home					
English	525 (92.8)	516 (92.8)	519 (93.2)	521 (93.7)	2081 (93.1)
Other	41 (7.2)	40 (7.2)	38 (6.8)	35 (6.3)	154 (6.9)
Health literacy (self-report)					
Inadequate	67 (11.8)	41 (7.4)	47 (8.4)	54 (9.7)	209 (9.4)
Adequate	499 (88.2)	515 (91.2)	510 (91.6)	502 (90.3)	2026 (90.4)
Health literacy (performance)					
High	269 (47.5)	266 (47.8)	267 (47.9)	273 (49.1)	1075 (48.1)
Medium	124 (21.9)	133 (23.9)	136 (24.4)	136 (24.5)	529 (23.7)
Low	173 (30.6)	157 (28.2)	154 (27.6)	147 (26.4)	631 (28.2)
Prior knowledge of health conditions					
Baseline sciatica knowledge (out of 4) mean (SD)	1.3 (0.8)	1.3 (0.7)	1.3 (0.8)	1.3 (0.7)	1.3 (1.0)
Baseline knee osteoarthritis knowledge (out of 4) Mean (SD)	2.0 (1.0)	2.0 (1.0)	2.0 (0.9)	2.0 (1.0)	2.0 (0.8)
Baseline total knowledge (out of 8)	3.3 (1.3)	3.3 (1.2)	3.3 (1.2)	3.2 (1.2)	3.3 (1.2)

Data displayed as n (%) unless otherwise indicated

### Primary Outcome

#### Knowledge

There was no evidence of a main effect of grade reading level on total knowledge after controlling for age, education, language spoken at home and baseline knowledge. There were no significant between-group differences in mean knowledge scores between the four groups (grade 8: 9.0 (SD:2.7), grade 10: 9.1 (SD:2.6), grade 12: 8.9 (SD: 2.6), grade 14: 9.1 (SD: 2.7) (Table 3). Multiple linear regression showed performance-based health literacy, self-reported prior knowledge and baseline knowledge to be significant predictors of overall knowledge scores (Table 4). There was no interaction between health literacy and grade reading level, therefore, it was excluded from the final model.

**Table 3:** Primary and Secondary Outcomes (Mean SD)

Variable	Grade 8 (*n* = 566)	Grade 10 (*n* = 556)	Grade 12 (*n* = 557)	Grade 14 (*n* = 556)	*p*-value
Knowledge					
Total knowledge *(out of 14)*	9.0 (2.7)	9.1 (2.6)	8.9 (2.7)	9.1 (2.7)	0.06
Validated knowledge *(out of 8)*	4.5 (1.6)	4.6 (1.5)	4.5 (1.5)	4.5 (1.5)	0.07
Perceived reading ease^*^ (out of 7)	5.8 (1.0)	5.7 (1.1)	5.6 (1.0)	5.7 (1.1)	0.14
Acceptability (out of 5)	4.2 (0.6)	4.1 (0.7)	4.2 (0.6)	4.2 (0.7)	0.09
Trustworthiness of information (out of 10)	8.4 (1.4)	8.3 (1.4)	8.4 (1.4)	8.4 (1.4)	0.60

**Table 4:** Unadjusted and Adjusted Linear Regression Models Predicting Total Knowledge Scores

	Unadjusted model			Adjusted model		
Predictor	Co-efficient	95% CI	*p*-value	Co-efficient	95% CI	*p*-value
Age	0.03	0.03 to 0.04	< 0.001	0.04	0.03 to 0.04	< 0.001
Education	−0.08	−0.15 to −0.00	0.048	−0.15	−0.22 to −0.09	< 0.001
*Language spoken at home*						
English	Reference					
Other	−0.55	−0.98 to −0.13	0.01	−0.25	−0.64 to 0.13	0.197
*Health literacy (performance based)*						
High	Reference					
Medium	−1.12	−1.39 to −0.85	< 0.001	−1.10	−1.36 to −0.84	< 0.001
Low	−2.19	−2.43 to 1.95	< 0.001	−2.09	−2.32 to −1.85	< 0.001
Baseline knowledge	0.19	0.12 to 0.26	< 0.001	0.22	0.15 to 0.28	< 0.001
*Self-reported knowledge of sciatica*						
High	Reference					
Low	0.62	0.38 to 0.86	< 0.001	0.29	0.05 to 0.53	0.018
*Self-reported knowledge of knee osteoarthritis*						
High	Reference					
Low	0.72	0.50 to 0.95	< 0.001	0.48	0.25 to 0.72	< 0.001
*Grade reading level*						
Grade 8	Reference					
Grade 10	0.08	−0.22 to 0.39	0.584	0.06	0.21 to 0.33	0.649
Grade 12	−0.24	−0.54 to 0.07)	0.127	0–0.17	−0.46 to 0.13	0.27
Grade 14	−0.07	−0.47 to 0.24	0.670	−0.08	−0.35 to 0.19	0.563

^*^ Two 7-point Likert scales for each topic (straightforward/confusing and easy/difficult) – all scores averaged, higher scores indicate higher perceived reading ease

### Secondary Outcomes

There were no significant differences in validated knowledge, perceived reading ease, acceptability or trustworthiness of information across the four grade reading levels. Perceived reading ease, acceptability and trustworthiness of information were relatively high for all four conditions (Table 3).

## DISCUSSION

This study used a randomised design to assess the impact of varying the grade reading level of written health information in isolation of other health literacy strategies. We found that reducing the grade reading level with minimal additional changes to text had no effect on consumer knowledge, perceived reading ease, acceptability or trustworthiness of information. Participants with higher health literacy were more likely to have higher knowledge scores overall compared to participants with medium and low levels of health literacy. Participants with low health literacy had similar knowledge scores across each of the four grade reading levels. This suggests that reducing the grade reading level alone may not be sufficient to reduce health literacy-related disparities.

Our findings extend the existing evidence base regarding the effect of health literate document design; however, they differ substantially from previous studies. Several previous studies found that simplifying health information such as informed consent forms and discharge summaries, resulted in improved comprehension.^19,20,23,35–38^ While some studies reduced the grade reading level of health information, they also employed a range of additional health literacy strategies. For example, a 2012 study by Benatar et al.^19^ found that a simplified informed consent form written at a grade 10 reading level resulted in higher comprehension scores compared to the standard consent form written at a grade 12 reading level. In addition to reducing the grade reading level, the authors also reported changing the content and adding diagrams and tables to the simplified form. Similarly, Davis et al. evaluated simplified vaccination information that was substantially shorter than the original version and included instructional graphics.^20^ Choudhry et al. found that simplified discharge summaries were associated with reduced telephone calls and readmission rates.^39^ However, the conclusiveness of the findings in this study are limited by the lack of randomisation.

Our findings may be partly explained by inherent limitations of readability formulas; they do not consider other elements that are important to comprehension such as word familiarity, prior experience of the reader and text cohesion.^40^ While previous studies evaluated complex interventions, we sought to explore the effect of changing readability alone. To do this, it was necessary to ignore other health literacy principles that contribute to accessibility and comprehension of written health information. In this sense, isolating the effect of readability is an artificial task. In reality, simplified materials are likely to incorporate a range of strategies.

### Strengths and Limitations

A major strength of our study was use of validated knowledge items. The use of a randomised design mitigates bias. Conducting the study online with a community sample had the advantage of being able to recruit a large sample size with enough statistical power to detect a small difference in knowledge scores. However, participants may have been less motivated to engage with the task due to the hypothetical nature of the information. This differs from previous studies that evaluated health texts in a clinical context where information was directly relevant to the sample population. Furthermore, we recognise the limitations of panel research. Panel members are often not representative of the general population. Conducting the trial in English and recruiting participants online is an important limitation. By limiting the sample to online participants, we potentially excluded participants with low computer literacy and digital health literacy. Only a small proportion of participants had less than high school education or spoke a language other than English at home. Despite using education quotas, 90% of participants were identified as having adequate health literacy using the single item literacy screener. While the lowest grade reading level tested in the present study was grade 8, it is possible that there may be a difference in outcomes with information written at lower levels (e.g., 5–6).

A strength of our study was the extensive piloting of the knowledge items with both experts and consumers to avoid floor and ceiling effects and avoid ambiguity in question responses. Holding all other elements constant and attempting to isolate the effect of grade reading level enabled us to gain a clearer understanding of the effect of grade reading level on knowledge. Another important strength is the use of methodologically rigorous readability formulas and evidence-based tools, giving us greater confidence in our findings.^10,11,27,31^

Revising health information to achieve readability thresholds at low grade reading levels is time and resource intensive and may result in a loss of information. This study suggests that there may be limited value in reducing grade reading level in isolation to mitigate disparities by health literacy. The benefits of reducing the grade reading level of written health information may be dependent on the inclusion of additional health literacy strategies and principles. More focus should be placed on plain language, optimising formatting and testing with consumers.

## CONCLUSION

Our findings support the recommendation for additional health literacy tools and strategies to be used alongside readability assessments when developing, revising and evaluating written health information. Readability scores should be interpreted with caution as a proxy for plain language. Further research is needed to determine appropriate, evidence-based target grade reading levels and to adequately determine the role of readability assessment in the context of health literate document design.
